# The cost-effectiveness of albumin in the treatment of decompensated cirrhosis in Germany, Italy, and Spain

**DOI:** 10.1186/s13561-019-0237-7

**Published:** 2019-07-05

**Authors:** M. Chris Runken, Paolo Caraceni, Javier Fernandez, Alexander Zipprich, Rashad Carlton, Martin Bunke

**Affiliations:** 1 Grifols Shared Services North America (SSNA), Inc., Research Triangle Park, Raleigh, NC USA; 2 Department of Medical and Surgical Sciences, Alma Mater Studiorum University of Bologna, Bologna, Italy; 3 Liver ICU, Liver Unit, Hospital Clinic, University of Barcelona, Barcelona, Spain; 4 European Foundation of Chronic Liver Failure (EF-Clif), Barcelona, Spain; 5 Department of Internal Medicine I, Martin-Luther-University Halle-Wittenberg, Halle, Germany; 6 Xcenda L.L.C, Palm Harbor, FL USA; 7 Senior Director Medical Affairs, Retrophin, San Diego, CA USA

**Keywords:** Albumin, Decompensated cirrhosis, Cost-effectiveness

## Abstract

**Background:**

Albumin is frequently prescribed in cirrhotic patients with acute decompensation. However, the true cost effectiveness of albumin use in cirrhotic patients is still under debate.

**Objective:**

To evaluate the cost-effectiveness of albumin in the treatment of decompensated cirrhosis in Germany, Italy, and Spain.

**Methods:**

A decision-tree economic model was developed to evaluate treatments for decompensated cirrhosis from the hospital perspective over a typical inpatient admission. The treatments for large volume paracentesis (LVP) were albumin vs saline, gelatin, or no fluid. The treatments for spontaneous bacterial peritonitis (SBP) were albumin plus antibiotics vs antibiotics alone. The treatments for hepatorenal syndrome (HRS) were albumin plus a vasoconstrictor vs a vasoconstrictor alone. Effectiveness inputs were literature-based. Cost inputs included pharmacy costs and medical complication costs of decompensated cirrhosis. The primary model assessments were incremental cost-effectiveness ratios (ICERs) per life saved and per quality-adjusted life-year (QALY).

**Results:**

Albumin was found to be both less costly and more effective relative to saline, gelatin, and no fluid for the treatment of LVP across all 3 countries. For SBP, albumin plus antibiotics was more clinically effective than antibiotics alone in all 3 countries. The combination of albumin plus antibiotics was less costly than antibiotics alone in Germany and Italy, making albumin a dominant treatment (ie, less costly and more effective). In the management of SBP in Spain, albumin plus antibiotics compared to antibiotics alone resulted in ICERs of €1516 per life saved and €3369 per QALY gained. Albumin plus a vasoconstrictor was both less costly and more effective than vasoconstrictor alone in the treatment of HRS across all 3 countries.

**Conclusion:**

This analysis demonstrates that albumin is cost-effective in terms of lives saved and QALYs gained in the management of decompensated cirrhosis associated with LVP, SBP, or HRS.

## Introduction

Cirrhosis is a chronic, severe clinical condition that can lead to the development of life-threatening complications as the disease progresses [1]. A recent report by the European Association for the Study of the Liver (EASL) shows that about 0.1% of the European population is affected by cirrhosis, leading to approximately 170,000 deaths per year (nearly 2% of all deaths in Europe) [2].

Depending on the absence or presence of clinically evident complications, cirrhosis is defined as being compensated or decompensated. Ascites, bleeding from gastro-esophageal varices, hepatic encephalopathy, and severe jaundice develop at a yearly rate of 5% to 7% and mark the transition to the decompensated stage [3]. Ascites is the most common and, often, the first complication of cirrhosis to appear, signaling the presence of decompensated cirrhosis [3]. While the median survival of compensated cirrhosis exceeds 12 years, survival drops to about 2 years after decompensation develops [3]. The pathophysiological scenario of decompensated cirrhosis presents 2 major systemic features: a circulatory dysfunction characterized by severe effective hypovolemia and a chronic inflammatory state. These alterations are inter-related and lead to the multi-organ dysfunction and failure occurring in end-stage cirrhosis [4]. The utilization of albumin to manage the complications of cirrhotic disease includes management of ascites with large volume paracentesis (LVP), spontaneous bacterial peritonitis (SBP), and hepatorenal syndrome (HRS).

Human albumin (HA) is currently given as treatment in patients with cirrhosis with the intent to counteract the effective hypovolemia based on its capacity to act as a plasma expander. Randomized clinical trials and meta-analyses have demonstrated the efficacy of HA to treat and prevent clinical complications of cirrhosis, which are characterized by effective hypovolemia [5–8]. International guidelines recommend the use of HA for the prevention of post-paracentesis circulatory dysfunction (PPCD) or renal failure induced by SBP and for the diagnosis and treatment of HRS in association with vasoconstrictor medications [9].

The removal of a large volume of ascitic fluid in LVP is associated with circulatory dysfunction, characterized by a reduction in effective blood volume, a condition known as PPCD [9]. To prevent circulatory dysfunction, the EASL guidelines recommend LVP along with the administration of 8 g of albumin per liter of ascitic fluid removed as first-line treatment in patients with large ascites not responding to diuretic therapy [9]. In patients undergoing LVP with removal of greater than 5 l of ascitic fluid, the use of plasma expanders other than albumin is not recommended because they are less effective in the prevention of PPCD [9].

SBP is defined as a bacterial infection of the ascitic fluid in the absence of a contiguous source of infection [10]. The prevalence of SBP is approximately 10% in inpatients with cirrhosis and 5% in outpatients with cirrhosis [11]. Despite treatment, deterioration of renal function, which has been reported in a third of patients with SBP, is a key predictor of in-hospital mortality [7]. The administration of albumin (1.5 g/kg at diagnosis and 1 g/kg on day 3) is recommended to decrease the frequency of HRS, thereby improving survival [9, 10].

HRS is defined as the occurrence of rapidly progressive functional renal failure in a patient with advanced liver disease in the absence of another identifiable cause [12]. The prognosis for HRS is poor, with only half of patients surviving at 1 month post-diagnosis [13, 14]. First-line treatment for type 1 HRS is terlipressin (1–2 mg/4–6 h by intravenous bolus or in continuous infusion) in combination with albumin [9].

Recent surveys in the United States and Europe have shown that the vast majority of physicians administer HA in patients presenting with these complications [15–17]. Nonetheless, concerns have been raised regarding the use of albumin in decompensated cirrhosis due to its perceived high cost [18]. However, the significant economic burden associated with these cirrhosis complications and their downstream healthcare costs, such as repeat hospitalizations, also have to be taken into account. Cost-effectiveness analyses of the use of albumin to treat complications of cirrhosis are lacking, which are critical for appropriate healthcare decision making. Therefore, the objective of this analysis is to evaluate the cost-effectiveness of albumin in the treatment of decompensated cirrhosis in Germany, Italy, and Spain.

## Methods

A decision-tree economic model was developed to evaluate the cost-effectiveness of various treatments for the complications of decompensated cirrhosis from the hospital perspective in Germany, Spain, and Italy. The model was developed using a 3-month time horizon, which was selected to best illustrate the hospital perspective and capture the length of an inpatient hospital stay for decompensated cirrhosis. The 3-month time horizon is based on a mean follow-up time of 76 days in the key meta-analysis for LVP and a follow-up duration of 3 months or until death or transplantation in the key trials for SBP and HRS [6–8]. Further, the 3-month time horizon was supported by European clinicians as reflecting the typical inpatient stay for decompensated cirrhosis. Effectiveness inputs in the model were literature-based and were applied to all 3 countries. The primary model assessments for each condition were incremental cost-effectiveness ratios (ICERs) per life saved and per quality-adjusted life-year (QALY).

### Large volume paracentesis

**Fig. 1:**
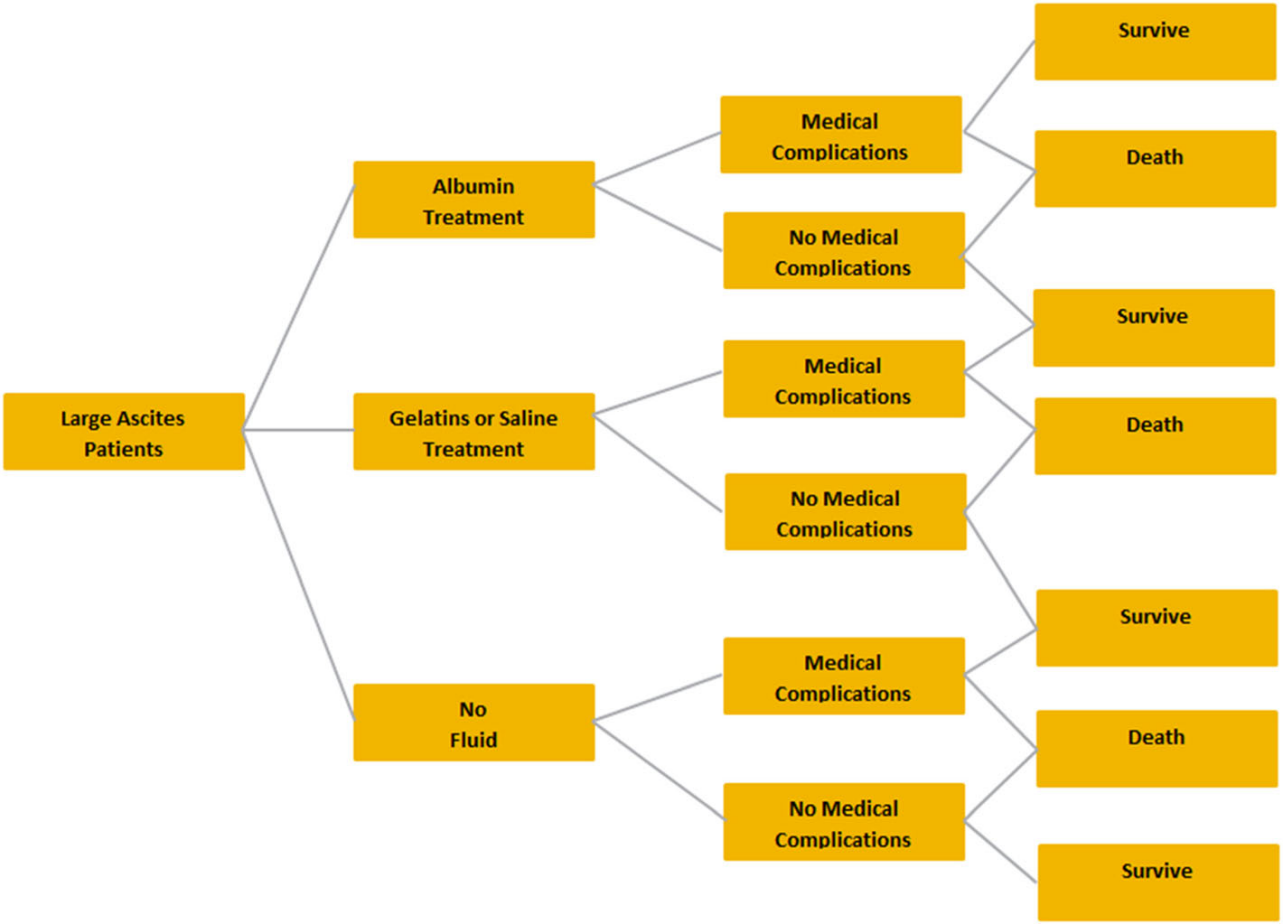
Decision tree for large volume paracentesis

The target LVP patient population is defined as those with ascites requiring greater than 5 l of ascitic fluid removal. The treatment strategies for LVP compared in the model included albumin vs saline, gelatin, or no fluid (Fig. 1). Hydroxyethyl starch (HES) solutions were not included, as their use is restricted by the European Medicines Agency. Effectiveness inputs used to compare the different therapeutic strategies included the rates of hyponatremia, renal impairment, hepatic encephalopathy (HE), and mortality. Hyponatremia was defined as a decrease in the serum sodium concentration of more than 5 mmol/L to a level below 130 mmol/L [19]. Renal impairment was defined as an increase in the serum creatinine concentration of more than 50% from the pretreatment value to a level greater than 133 μmol/L (1.5 mg/dL) [19]. HE was defined as grade 2 to 4 (moderate to severe) [19]. The treatment strategies and effectiveness inputs were selected to reflect the current treatment practice in Europe and were validated by a group of European clinicians.

Pharmacy costs for LVP and inpatient medical costs for the complications of hyponatremia, renal dysfunction, and HE were considered in model comparisons. Costs in the model reflect 2017 costs. Pharmacy doses were based upon the average amount of fluid removed (8.3 L), and the dose of each plasma volume expander was based on a meta-analysis of patients in randomized clinical trials undergoing LVP [6]. The average pharmacy dose was 8 g/L for albumin, 150 mL/L for gelatin, and 170 mL/L for saline [9, 20]. The pharmacy and medical complication costs were specific to each country.

LVP QALYs were calculated based on the 2004 Wells article defining health state utilities for decompensated cirrhosis (0.74) and HE with decompensated cirrhosis (0.55) [21]. Patients with cirrhosis receiving LVP who survive are assumed to have the health state utility for decompensated cirrhosis (0.74), with the exception of patients who experience HE, who are assumed to have the health state utility value for HE (0.55).

### Spontaneous bacterial peritonitis

**Fig. 2:**
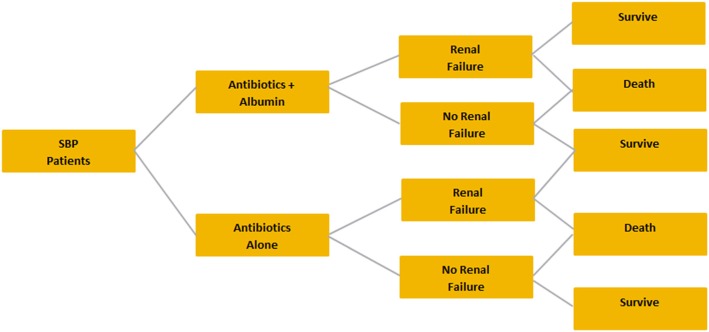
Decision tree for spontaneous bacterial peritonitis

The target population for SBP was patients with decompensated cirrhosis presenting with SBP. The treatment strategies for SBP compared in the model were albumin plus antibiotics vs antibiotics alone (Fig. 2). Effectiveness inputs included renal impairment rates, length of hospital stay, and mortality. Renal impairment was defined as a nonreversible deterioration of renal function during hospitalization [7]. In patients without renal failure at enrollment, renal impairment was diagnosed when the blood urea nitrogen or serum creatinine level increased by more than 50% of the pretreatment value to levels higher than 30 mg/dL or 1.5 mg/dL, respectively [7]. In patients with preexisting renal failure, an increase in blood urea nitrogen or serum creatinine level by more than 50% from baseline was required for a diagnosis of renal impairment [7].

Pharmacy costs for SBP, inpatient medical costs for the complication of renal impairment, and length of hospital stay costs were all considered in model comparisons. The pharmacy dose for antibiotics was 8 g of cefotaxime per day (2 g intravenously every 6 h) for 5 days and 100 g on day 1 (1.5 g/kg at diagnosis, maximum of 100 g) plus 70 g on day 3 (1 g/kg) for albumin [9]. The average patient weight was assumed to be 70 kg.

QALYs for SBP were calculated based on the health state utility value for SBP (0.45) [21]. Patients who survived 3 months were assumed to have the health state utility value for SBP.

### Hepatorenal syndrome

**Fig. 3:**
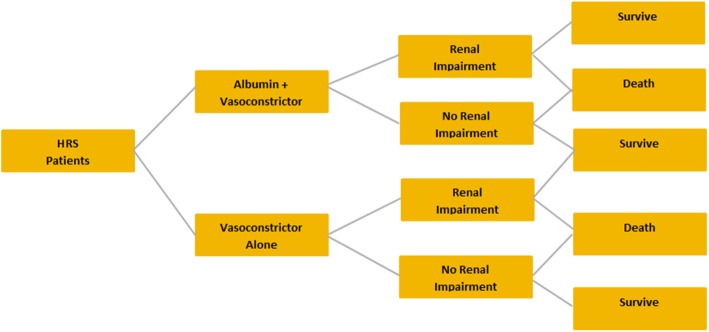
Decision tree for hepatorenal syndrome

The target patient population for HRS was patients with decompensated cirrhosis who developed type 1 HRS. The modeled therapeutic strategies for HRS were albumin plus a vasoconstrictor vs that vasoconstrictor alone (Fig. 3). Vasoconstrictors used in the modeling exercises, as advised by European clinicians, were either terlipressin or noradrenaline.

Effectiveness inputs included renal impairment and mortality. Response to treatment was defined by a decrease in serum creatinine to ≤1.5 mg/dL (complete response) or ≥ 50% decrease in serum creatinine to > 1.5 mg/dL (partial response) [8]. Patients who did not achieve a complete or partial response were assumed to have renal impairment, with the rate of renal impairment was calculated as 1 minus the percentage of patients with improved renal function.

Pharmacy costs for HRS and inpatient medical costs for complications of renal impairment were considered in model comparisons. The total pharmacy dose was 235 g of albumin (1 g/kg on day 1 and 20–40 g/day thereafter until reversal of HRS or a maximum of 15 days) and 44 mg of terlipressin (1 mg every 4–6 h, increased to a maximum of 2 mg every 4–6 h) for the combination and 34 mg for terlipressin alone [9, 22]. The total pharmacy dose was 182 g of albumin (20 g/day, for a mean of 9.1 days) and 119 mg for noradrenaline (13.1 mg/day, for a mean of 9.1 days) for the combination and 119 mg for noradrenaline alone [9, 23].

HRS QALYs were calculated based on the health state utility value for decompensated cirrhosis (0.74) [21].

The decision trees for LVP, SBP, and HRS are included in Fig. 1. The country-specific cost inputs and their references, as well as the effectiveness inputs and the referencing materials used for their model calculations, are included in Tables 1 and 2, respectively.

**Table 1:** Country-Specific Cost Inputs

Cost Input	Germany	Italy	Spain
Pharmaceutical costs			
Albumin (g)	€4.68 [24]	€4.35 [25]	€3.69 [26]
Gelatin (100 mL)	€1.61 [24]	€0.67 [25]	€1.47 [26]
Saline (100 mL)	€0.33 [24]	€0.36 [25]	€0.40 [26]
Antibiotics (g) (cefotaxime)	€6.38 [24]	€3.40 [25]	€3.12 [26]
Terlipressin (mg)	€54.55 [24]	€19.41 [25]	€16.38 [26]
Noradrenaline (mg)	€0.61 [24]	€0.32 [25]	€0.47 [26]
Medical costs			
Renal impairment	€14,178 [27]	€5329 [28]	€4089 [29]
Hepatic encephalopathy	€18,134 [27]	€13,393 [30]	€3190 [29]
Hyponatremia	€2203 [31]	€3000 [assumption]	€4023 [32]
Hospital inpatient day	€794.94 [27]	€397.21 [30]	€601.22 [33]

**Table 2:** Effectiveness and Utility Inputs^a^

Treatment	Hyponatremia Incidence (%)	Renal Impairment Incidence (%)	HE Incidence (%)	Hospital Length of Stay (Days)	Mortality (%)	Utility Values
LVP after ascites						
Albumin	8.8% [6]	7.2% [5]	3.1% [5]	–	2.1% [5]	–
Gelatin	22.6% [6]	10.1% [5]	5.1% [5]	–	6.1% [5]	–
Saline	14.3% [20]	8.6% [20]	5.4% [5]^b^	–	2.9% [20]	–
No fluid	16.5% [6]	11.3% [34]	5.7% [34]	–	3.8% [34]	–
Decompensated cirrhosis	–	–	–	–	–	0.74
Decompensated cirrhosis with encephalopathy	–	–	–	–	–	0.55
SBP						
Antibiotics + albumin	–	10% [7]^e^	–	14 [7]^e^	22% [7]	–
Antibiotics alone	–	33% [7]^e^	–	13 [7]^e^	41% [7]	–
Spontaneous bacterial peritonitis	–	–	–	–	–	0.45
HRS						
Albumin + terlipressin	–	29.6% [8]^c^	–	–	40.7% [8]	–
Albumin + noradrenaline	–	52.4% [23]^c^	–	–	52.4% [23]	–
Vasoconstrictor alone^d^	–	75.0% [22]^c^	–	–	87.5% [22]^h^	–
Decompensated cirrhosis	–	–	–	–	–	0.45

^a^Data reflect the rates of various complications as reported in the literature

^b^The rate of HE in patients treated with saline was assumed to be the same as the HE rate for those treated with dextran

^c^The data reflect the percentage of patients without resolution of their renal impairment

^d^The effectiveness inputs for vasoconstrictor alone are based on terlipressin results

Key: *HE* hepatic encephalopathy, *HRS* hepatorenal syndrome, *LVP* large-volume paracentesis, *SBP* spontaneous bacterial peritonitis

## Results

### Large volume paracentesis

**Fig. 4:**
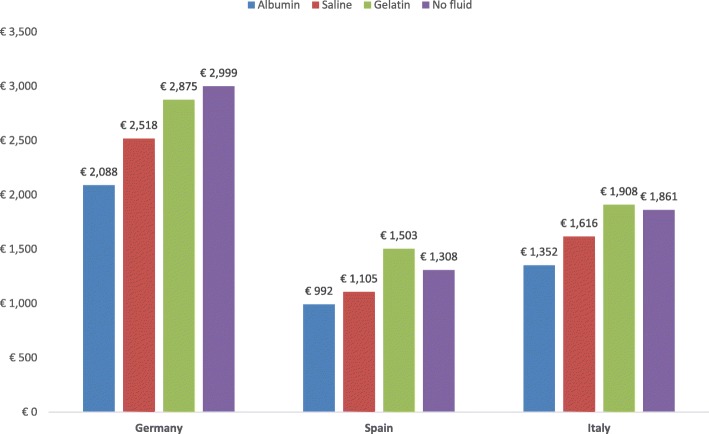
Expected cost of treatment of the different strategies for large volume paracentesis

**Fig. 5:**
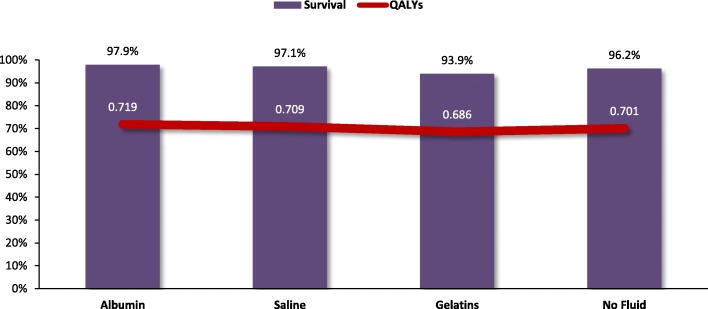
Quality-adjusted life-year and survival of the different strategies for large volume paracentesis

The total calculated treatment cost per patient with ascites undergoing LVP was lower with albumin treatment than with saline, gelatin, or no fluid in Germany, Italy, and Spain (Fig. 4). These lower total costs were driven by lower medical complication costs for hyponatremia, renal impairment, and HE. Treatment with albumin over the 3-month time horizon also led to lower mortality and fewer HE complications, resulting in more QALYs gained compared with saline, gelatin, or no fluid (Fig. 5). Since albumin was both less costly and more effective (ie, a dominant treatment) relative to saline, gelatin, and no fluid across all 3 countries, individual ICERs did not need to be calculated.

### Spontaneous bacterial peritonitis

**Fig. 6:**
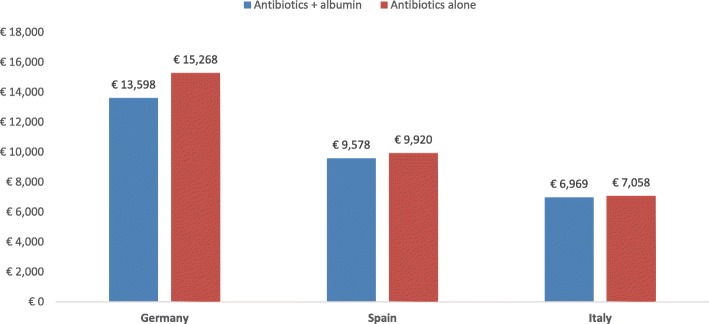
Expected cost of treatment of the different strategies for SBP

**Fig. 7:**
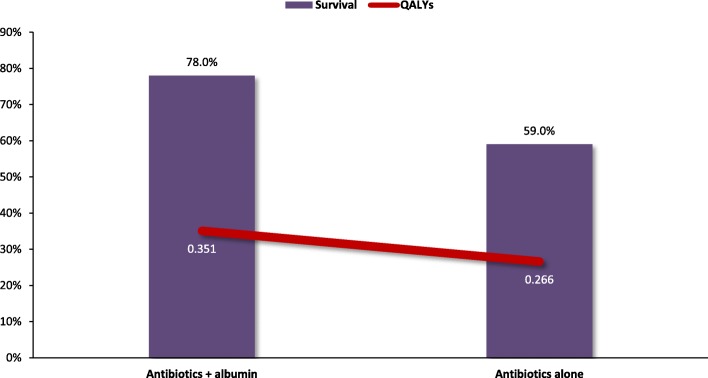
Quality-adjusted life year and survival of the different strategies for SBP

The total computed healthcare cost per SBP patient was lower when they were treated with antibiotics plus albumin than antibiotics alone in Germany and Italy, but not in Spain (Fig. 6). Since the same effectiveness data were used in each country, treatment with antibiotics plus albumin resulted in lower mortality (22% vs 41%) and higher QALYs gained (0.351 vs 0.266) in all 3 countries (Fig. 7). However, since cost savings occurred in Germany and Italy, treatment with antibiotics plus albumin was a dominant treatment, eliminating the need for ICER calculations. Since the total costs for antibiotics plus albumin treatment were higher in Spain, ICER values were calculated, resulting in costs of €1516 per life saved and €3369 per QALY gained for the Spanish system.

### Hepatorenal syndrome

**Fig. 8:**
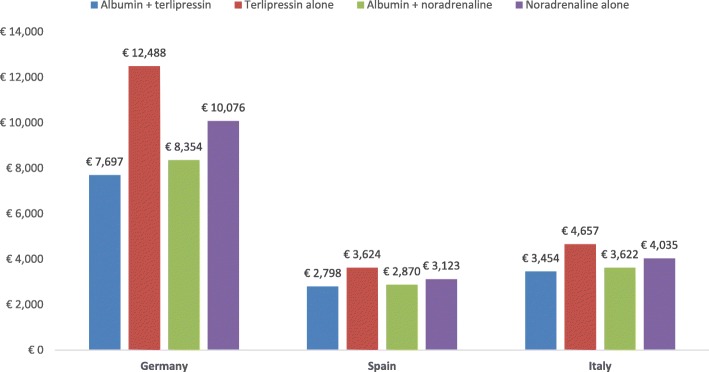
Expected cost of treatment of the different strategies for hepatorenal syndrome

The total cost per HRS patient was lower with albumin plus terlipressin treatment than with terlipressin alone in all 3 countries, mostly due to lower medical complication costs. Similarly, the total cost per patient was lower with albumin plus noradrenaline than with noradrenaline alone (Fig. 8). Treatment with albumin plus a vasoconstrictor was less costly than a vasoconstrictor alone in all 3 countries, mostly due to the lower medical complication costs.

**Fig. 9:**
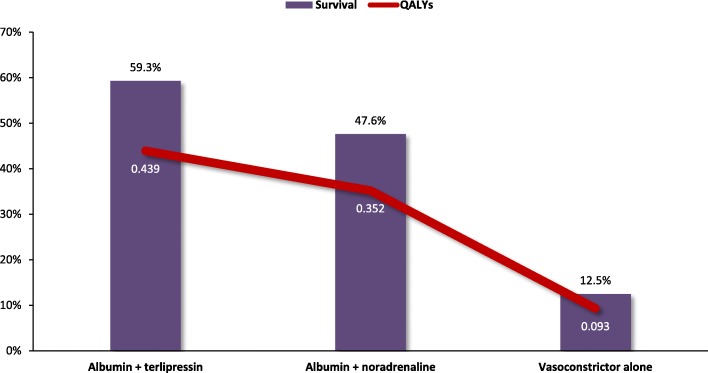
Quality-adjusted life-year and survival of the different strategies for hepatorenal syndrome

Treatment with albumin plus terlipressin resulted in more QALYs gained than terlipressin alone (0.439 vs 0.093). Since albumin plus terlipressin was the dominant therapy, individual ICERs were not calculated. Treatment with albumin plus noradrenaline also resulted in more QALYs gained than noradrenaline alone (0.352 vs 0.093) (Fig. 9). Individual ICERs were not calculated for albumin plus noradrenaline either, as it too was a dominant therapy.

### Sensitivity analysis

A probabilistic sensitivity analysis was conducted to address uncertainty in the model. For each condition, the costs inputs, effectiveness inputs, and utility values were sampled for 1000 simulations. The sensitivity analysis results are presented using Spain as the base case country. For LVP, albumin was cost-effective compared to saline, gelatins, and no fluid 100% of the time at a willingness to pay value greater than €0/QALY. For SBP, antibiotics plus albumin were cost-effective compared to antibiotics alone 69% of the time at willingness to pay of €0/QALY, 99.4% of the time at €25,000/QALY, and 100% of the time at a willingness to pay greater than €30,000/QALY. For HRS, albumin plus terlipressin was cost-effective compared to terlipressin alone 100% of the time at a willingness to pay value greater than €0/QALY.

## Discussion

This analysis evaluated the theoretical cost-effectiveness of albumin in the treatment of decompensated cirrhosis requiring LVP, with SBP, or with HRS, across 3 European countries, Germany, Italy, and Spain, from a hospital perspective. With the model evaluating the cost-effectiveness from a hospital perspective, a decision-tree model was selected to represent the clinical pathways for the inpatient portion of treatment for decompensated cirrhosis. The decision-tree methodology allowed for the synthesis of evidence from multiple clinical trials conducted in Europe with albumin and multiple comparators to evaluate the cost-effectiveness of albumin in the treatment of decompensated cirrhosis requiring LVP with SBP or with HRS.

In the LVP analyses, the total cost of treatment with albumin was both less costly and more effective than saline, gelatin, or no fluid. The higher pharmacy costs with albumin were offset by the cost savings of reduced medical complication rates, resulting in lower total costs of therapy in cirrhosis patients treated with albumin. The lower mortality rates associated with albumin compared to saline, gelatin, and no fluid resulted in higher QALYs gained, demonstrating both the economic and humanistic benefits of albumin in the treatment of LVP.

In the SBP treatment analysis, the use of albumin in addition to antibiotics in Germany and Italy resulted in lower total costs than treatment with antibiotics alone, in addition to being more effective than antibiotics alone, resulting in a dominant role for albumin. However, in Spain, where pharmacy and medical complication costs are both lower, treatment with albumin plus antibiotics resulted in slightly higher total treatment costs than antibiotics alone (€288 difference). The improved survival and increase in QALYs gained with the combination of albumin plus antibiotics in Spain resulted in ICERs below the commonly accepted cost-effectiveness thresholds [35, 36]. The results observed in our model for SBP patients were similar to those shown in an analysis from Farrugia et al., where the use of albumin with antibiotics resulted in lower costs, improved survival, additional QALYs, and lower costs per QALY gained [37].

In the HRS analyses, the total cost of therapy with albumin plus a vasoconstrictor was less than with a vasoconstrictor alone due to reduced rates of renal impairment. Albumin plus a vasoconstrictor also decreased mortality, resulting in more QALYs gained.

In Germany the use of albumin is recommended in the national guidelines for these specific treatments. Besides this fact, adherence to these treatments is not complete due to the perceived cost of albumin. The results of this study show that the treatment with albumin is cost effective. The cost of albumin in our study in Germany was fairly high. However, a lower price of albumin would even increase the cost effectiveness, a finding that should be a strong argument to use albumin according to the international and national guidelines.

In Spain, albumin administration is widely used in the 3 clinical decompensations evaluated in this cost-effectiveness analysis following national and international guidelines. Its clinical efficacy is considered by many physicians a key determinant to prescribe this drug beyond its economic cost. The current study suggests that besides improving survival and QALYs, albumin administration is cost-effective in the majority of the clinical scenarios in which it is prescribed nowadays.

In Italy, the use of albumin is recommended by national guidelines to treat or prevent severe complications of cirrhosis such as circulatory dysfunction after LVP, renal failure caused by SBP, and treatment of HRS along with vasoconstrictors. However, albumin is underutilized mainly due to its higher pharmacy cost vs other fluids, leading to health authorities and hospital administrations restricting its use. The results of this study show that when considering total cost of therapy in addition to improving survival and quality of life, albumin is cost-effective and should be used in accordance with guideline recommendations in Italy.

As a decision-tree economic model, this analysis presents certain limitations. The efficacy and cost data used in the model were taken from various studies with different patient populations. However, efforts were made to use current studies with similar designs and to take efficacy inputs from comparable trials. Dosing in the analysis was based on clinical guidelines, where available, to reflect the current dosing for decompensated cirrhosis. As such, dosing was not country-specific and may not reflect differences in treatment decisions made by physicians. However, physicians from the 3 country-specific regions were consulted for this manuscript. For the LVP study with saline, 3% saline was used in the trial, but the costs in the analysis reflect the more commonly used 0.9% normal saline [20].

Costs for medical complications in cirrhosis were based on the cost of treating the condition of interest (eg, hyponatremia, renal impairment, HE), but they were not always specific to patients with the exact condition and cirrhosis. Furthermore, costs were based on publicly available data sources and may not reflect the actual amount paid by payers in the respective countries for individual patient cases due to interpatient variability. The rates of medical complications were based on the rates reported in the individual studies, which may have used slightly different definitions for each condition. Every attempt was made to select studies with comparable medical complication definitions and disease severity.

## Conclusions

The results of this analysis demonstrate that the use of albumin in the management of decompensated cirrhosis associated with LVP, SBP, or HRS results in lower total costs and improved clinical outcomes compared to other fluids. Furthermore, the results show that albumin is cost-effective in terms of lives saved and QALYs gained across Germany, Spain, and Italy. Therefore, albumin should be considered as a first-line treatment option in cirrhotic patients with these clinical decompensations.

## Data Availability

All relevant data are contained within the results. Input data were derived from the listed references.

## Abbreviations

EASL: European Association for the Study of the Liver
HA: Human albumin
HE: Hepatic encephalopathy
HES: Hydroxyethyl starch
HRS: Hepatorenal syndrome
ICER: Incremental cost-effectiveness ratio
LVP: Large volume paracentesis
PPCD: Post-paracentesis circulatory dysfunction
QALY: Quality-adjusted life-year
SBP: Spontaneous bacterial peritonitis
