# Correction to: The cost-effectiveness of albumin in the treatment of decompensated cirrhosis in Germany, Italy, and Spain

**DOI:** 10.1186/s13561-020-00265-0

**Published:** 2020-04-17

**Authors:** M. Chris Runken, Paolo Caraceni, Javier Fernandez, Alexander Zipprich, Rashad Carlton, Martin Bunke

**Affiliations:** 1 Grifols Shared Services North America (SSNA), Inc, Research Triangle Park, Raleigh, NC USA; 2 Department of Medical and Surgical Sciences, Alma Mater Studiorum University of Bologna, Bologna, Italy; 3 Liver ICU, Liver Unit, Hospital Clinic, University of Barcelona, Barcelona, Spain; 4 European Foundation of Chronic Liver Failure (EF-Clif), Barcelona, Spain; 5 Department of Internal Medicine I, Martin-Luther-University Halle-Wittenberg, Halle, Germany; 6 Xcenda L.L.C, Palm Harbor, FL USA; 7 Senior Director Medical Affairs, Retrophin, San Diego, CA USA

**Correction to: Health Economics Review (2019) 9:22**

**https://doi.org/10.1186/s13561-019-0237-7**

Following publication of the original article [1], the authors reported that one of the numbers within Fig. 6 contains a mistake.

The correct Fig. 6 is shown below.

**Fig. 6:**
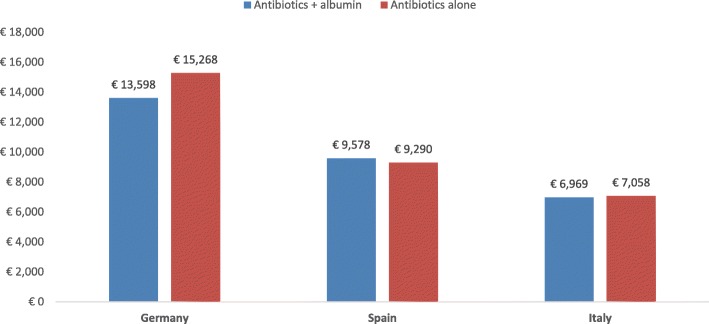
Expected cost of treatment of the different strategies for SBP
